# The prevalence of autism spectrum traits and autism spectrum disorders in children and adolescents with obsessive compulsive disorder: a systematic review

**DOI:** 10.1192/bjo.2021.194

**Published:** 2021-06-18

**Authors:** Claire Tiley, Marinos Kyriakopoulos

**Affiliations:** 1 South London and Maudsley NHS Foundation Trust; 2 South London and Maudsley NHS Foundation Trust, Institute of Psychiatry, Psychology and Neuroscience, King's College London

## Abstract

**Aims:**

Autism Spectrum Disorder (ASD) and Obsessive Compulsive Disorder (OCD) commonly co-occur in children and adolescents (C&A); evidence suggests functional impairment is increased in those diagnosed with both disorders. The aims of this systematic review were: 1) To review studies that report on the prevalence of ASD traits and/or diagnosis in C&A with OCD. 2) To review whether the severity of OCD symptoms is related to the severity of ASD traits in C&A with OCD. 3)To review whether the severity of comorbid ASD traits or diagnosis in C&A with OCD impact on their global functioning.

**Method:**

This systematic review was registered in PROSPERO. Prisma guidelines were followed . Electronic searches were carried out on Pubmed, EMBASE and Psychinfo with the use of selected keywords. Inclusion criteria : 1) Participants up to the age of 18 who had an ICD or DSM diagnosis of OCD. 2) Journal articles published in the English, with no date specifications. 3) Papers evaluating ASD diagnosis or traits, or where data on this could be extracted. Exclusion criteria: 1) Papers looking at OCD related disorders such as body dysmorphic disorder, compulsive skin picking, trichotillomania and hoarding disorder. 2) Samples including adults where C&A data could not be extracted. 3) Posters, abstracts and dissertations.

**Result:**

A total of 15 studies were included in the systematic review. Seven of these studies directly compared the prevalence of ASD traits (measured by questionnaires) or diagnosis in OCD to a control group or normative data, with all studies reporting a significant elevation in ASD trait scores and diagnosis in OCD. Ten of the studies reported on the correlation between ASD trait severity and OCD severity. Four studies identified a significant correlation between ASD and OCD total scores or specified subscales. In contrast, one study found significantly elevated OCD scores in an OCD only group when compared to a comorbid OCD and ASD group. Three studies reported on the correlation between ASD scores and functional impairment or compared an OCD only group to a comorbid group. All three studies demonstrated that the presence ASD or ASD traits are associated with elevated scores in global functional impairment.

**Conclusion:**

In conclusion, this review suggests that there is an increased prevalence of ASD traits and diagnosis amongst C&A with OCD. Elevated ASD traits within this population are associated with a greater impact on global functioning.

